# Supplementary material for: Investigation of the Mechanism of Action of Periploca forrestii Schltr. Extract on Adjuvant Collagen Rats Based on UPLC-Q-Orbitrap-HRMS Non-Targeted Lipidomics
Source: Molecules. 2023 Sep 22;28(19):6751. doi: 10.3390/molecules28196751 (PMC10574421; doi:10.3390/molecules28196751)
Supplement: Supplementary file 1 [file molecules-28-06751-s001.zip › supplementary material/Figure S1-S4.docx]

Total ionogram of positive and negative ion patterns in serum and urine of AA rats.

Figure S1 Total Serum Positive Ion Flow Diagram

Figure S2 Total Serum Negative Ion Flow Diagram

Figure S3 Urine positive total ion flow diagram

Figure S4 Urine negative total ion flow diagram
